# A feasibility study of multi-site,intracellular recordings from mammalian neurons by extracellular gold mushroom-shaped microelectrodes

**DOI:** 10.1038/srep14100

**Published:** 2015-09-14

**Authors:** Silviya M. Ojovan, Noha Rabieh, Nava Shmoel, Hadas Erez, Eilon Maydan, Ariel Cohen, Micha E. Spira

**Affiliations:** 1 Department of Neurobiology, The Alexander Silberman Institute of Life Science, and the Harvey M. Kruger family Center for Nanoscience. The Hebrew University of Jerusalem, Edmond J. Safra Campus, Jerusalem, 91904, Israel; 2 Intel Israel LTD, S.B.I. Park Har Hotzvim, CFF8, Jerusalem 91031, Israel

## Abstract

The development of multi-electrode array platforms for large scale recording of neurons is at the forefront of neuro-engineering research efforts. Recently we demonstrated, at the proof-of-concept level, a breakthrough neuron-microelectrode interface in which cultured *Aplysia* neurons tightly engulf gold mushroom-shaped microelectrodes (gMμEs). While maintaining their extracellular position, the gMμEs record synaptic- and action-potentials with characteristic features of intracellular recordings. Here we examined the feasibility of using gMμEs for intracellular recordings from mammalian neurons. To that end we experimentally examined the innate size limits of cultured rat hippocampal neurons to engulf gMμEs and measured the width of the “extracellular” cleft formed between the neurons and the gold surface. Using the experimental results we next analyzed the expected range of gMμEs-neuron electrical coupling coefficients. We estimated that sufficient electrical coupling levels to record attenuated synaptic- and action-potentials can be reached using the gMμE-neuron configuration. The definition of the engulfment limits of the gMμEs caps diameter at ≤2–2.5 μm and the estimated electrical coupling coefficients from the simulations pave the way for rational development and application of the gMμE based concept for in-cell recordings from mammalian neurons.

The development of multi-electrode array (MEA) platforms for large scale, long-term recording and stimulation of neurons under *in vitro* and *in vivo* conditions is at the forefront of neuroscience research and development^1^,^2^,^3^. Although recent research and development efforts have successfully focused on increasing the number, density and spatial organization of MEA^4^,^5^,^6^, they still suffer from a low signal to noise ratio and limited source separation. Individual sensors pick up extracellular field potentials generated by a number of neurons and require elaborate spike detection and sorting^7^. Worse, current *in vitro* and *in vivo* MEAs are totally blind to synaptic and subthreshold membrane oscillations generated by single neurons^8^. Synaptic potentials can only be recorded if generated synchronously by populations of neurons^9^. To overcome these limitations, a number of laboratories have begun to develop new approaches to enable simultaneous electrical recordings from many individual neurons at a quality comparable to intracellular recordings by sharp or patch electrodes^8^,^10^,^11^,^12^. These efforts include the development of single or multi electrode platforms which utilize nanometer scale structures that gain direct Ohmic contact with the cytosol by penetrating the plasma membrane of the cells in a way similar to classical sharp electrodes^13^,^14^,^15^,^16^,^17^,^18^,^19^,^20^,^21^ or by membrane electroporation^22^,^23^,^24^. With the exception of nano-transistors which record full-blown action potentials when introduced into cultured cardiomyocytes^10^,^11^,^20^, passive nanoelectrodes (nanopillars) record attenuated action potentials with amplitudes in the averaged range of single millivolts^16^,^19^,^22^,^23^.

In recent studies our laboratory has developed a different approach dubbed “IN-CELL recording” in which micrometer-sized, extracellular gold mushroom-shaped microelectrodes (gMμEs) record attenuated synaptic and action potentials with the characteristic features of intracellular recordings^8^,^25^,^26^,^27^,^28^,^29^,^30^. In these proof-of-concept studies we demonstrated that cultured *Aplysia* neurons tightly engulf gMμEs to form a high seal resistance (*R*_*s*_). This, together with increased conductance of the neuronal membrane that faces the electrode (the junctional membrane - *jm*), makes it possible to record action potentials and subthreshold synaptic potentials with qualities and biophysics similar to perforated patch recordings^31^. Ultrastructural studies have revealed that various cell types including NIH/3T3, CHO, PC-12, H9C2, HL-1 cell lines as well as primary cultured rat hippocampal neurons also engulf mushroom shaped electrodes^25^,^26^,^27^,^30^,^32^,^33^,^34^, thus suggesting that the cell-biological mechanisms leading to gMμE engulfment may be ubiquitous.

Historically the micrometer size and shape of the gMμEs used in our laboratory was selected by mimicking the dimensions and shape of the post synaptic spine structures that extend from the dendrites of vertebrate neurons^26^,^35^. Nevertheless, from a practical neuroengineering point of view, larger diameter gMμEs are expected to provide better electrical coupling.

Although no studies have attempted to optimize IN-CELL recordings by increasing the size of gMμEs, several laboratories have explored the potential use of nanometric size mushroom shaped electrodes^32^,^34^,^36^. These studies are based on the reasoning that nanometric sized mushroom-shaped microelectrodes may be more suitable for interfacing with small (10–20 μm diameter) mammalian neurons than the very large *Aplysia* neurons (50–80 μm diameter) used in our proof-of-concept studies. These studies showed that approximately 500 nm mushroom shaped protrusions are engulfed by cultured HL-1 cells and rat hippocampal neurons. Nevertheless, in these studies the recorded field potentials lacked the features of IN-CELL recordings. Rather, they were characterized by being similar to classical extracellular recordings of biphasic or monophasic negative field potential with small amplitudes of 100–200 μV^32^,^34^.

The above background implies that to improve the electrical coupling between small mammalian neurons and extracellular gMμEs for noninvasive long-term intracellular recordings and stimulation, larger rather than smaller gMμEs should be considered, or other characteristics of the gMμE should be improved.

So far, no study has attempted to quantitatively estimate the optimal gMμEs size to obtain the maximal range of electrophysiological signaling amplitudes generated by mammalian neurons. Optimization of the neuron-gMμE coupling coefficient depends on three classes of parameters: (a) the innate cell biological mechanisms that limit the gMμE-cap diameter that can be totally engulfed by a mammalian neuron. (b) The cleft width formed between the neuron’s membrane and the surface of the gMμE. (c) The size- and material-dependent electrical parameters of the gMμE. In the present study we examined these questions. Based on the experimental results we quantitatively estimated the expected levels of mammalian neurons-gMμEs coupling coefficients. The biological examination revealed that the size limits of the gMμEs cap that can be effectively engulfed by hippocampal neurons is 2–2.5 μm. Beyond this diameter the neurons can adhere to the upper surface of the mushrooms cap but fail to engulf it. Computer simulations of the neuron-gMμE configuration, which took into account the limited size of the mushroom cap and various structural and electrical parameters, then provided the range of electrical coupling coefficient that can be expected from the mammalian neuron-gMμE configuration. The findings presented here constitute a bioengineering framework for the rational design, development and application of gMμEs based platforms for IN-CELL recordings from mammalian neurons.

## Results

### Structural interfacing between neurons and protruding gold mushroom-shaped microelectrodes

**Figure 1:**
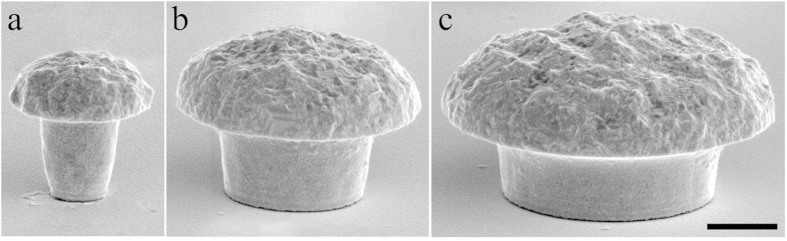
Scanning electron microscope images of gold mushroom-shaped protrusions (gMμP) that were fabricated to examine the structural interface between cultured rat hippocampal neurons and the protruding microstructures. (**a**) Small, (**b**) medium and (**c**) large gMμPs with 1.5–2, 3–3.5 and 4–5 μm cap diameters, respectively. Note the rough surface of the mushroom caps. Calibration −1 μm.

To define the largest gMμE size that can be engulfed by mammalian neurons we cultured dissociated 17 day old rat embryonic (E17) hippocampal neurons^37^ on matrices of gold mushroom-shaped protruding micro-structures (gMμP) of different sizes. The E17 culturing procedure yields a culture enriched by neuronal cells with only a few glial cells and thus can be used to examine the ultrastructural relationships between neurons and gMμPs (Supplementary Fig. 1). The neurons were cultured on three matrices made of small, medium and large gMμPs with cap diameters of 1.5–2, 3–3.5 and 4–5 μm and stalk diameters of 1, 2 and 3 μm, respectively (Fig. 1). Because the thickness of the cell body cytoplasm of cultured rat hippocampal neurons is in the range of 1–2 μm and the fact that mechanical deformation of the nuclear envelop may alter gene expression^38^,^39^, we kept the height of the gMμP at 1.5–2 μm (1–1.3 stalk and 0.5–0.7 μm cap heights, respectively). The center to center spacing between the micro-protrusions was adjusted to generate spaces of 8 μm between the perimeters of the mushroom caps. This spacing was selected to increase the probability for thin sections prepared for transmission electron microscopic (TEM) observation to run through the gMμP but still avoid inhibition of the electrode engulfment as a result of overly densely spaced microstructures^40^,^41^. The entire surface of the matrices was functionalized by PDL and laminin.

**Figure 2:**
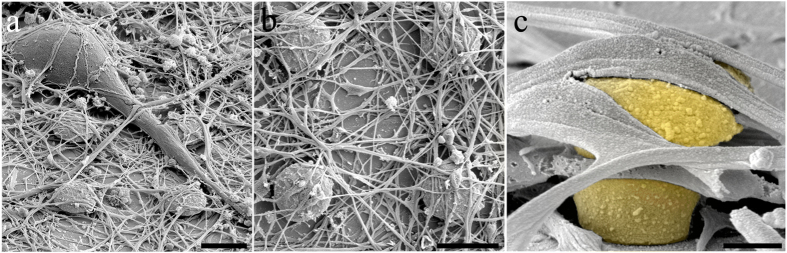
Scanning electron microscope images of neurons grown on a matrix of gMμPs. (**a**) A low magnification image of a neuron’s cell body and an extending neurite on a matrix of large gMμPs. (**b**) Neurites growing on top of large gMμPs as well as on the flat substrate in between the microprotrusions. (**c**) Neurites that extend on top of a mushroom cap appear to tightly adhere to the gold surface (labeled yellow) of a small gMμP. Calibration bars: 5 μm for (**a**,**b**), and 0.5 μm for (**c**).

Scanning electron microscopy (SEM) of the cultures revealed that independent of the gMμP cap diameter, cell bodies and neurites adhere to the flat substrate in between the microprotrusions and to the caps or stalks of the gMμPs (Fig. 2).

**Figure 3:**
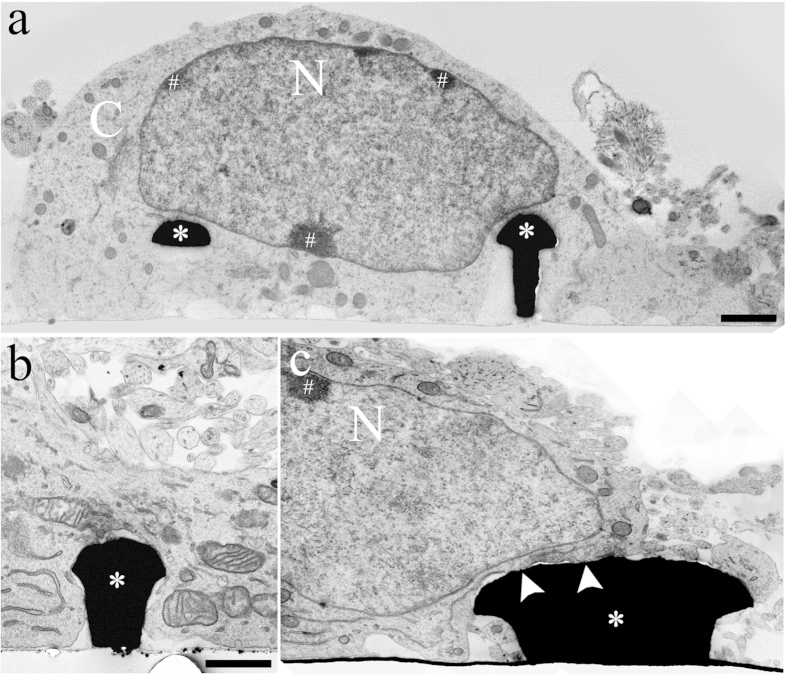
Transmission electron microscope (TEM) images of cultured hippocampal neurons engulfing gMμPs. (**a**) Low magnification of a neuron’s cell body engulfing two small gMμPs. On the left hand side the section went through a gold cap. On the right hand side the section went through the stalk and the cap. Note the slightly depressed nuclear membrane in the vicinity of the gMμP. (**b**) Tight engulfment of a small gMμP by a thick neurite. (**c**) An example of the interface between a cell body and the cap of a large gMμP. Calibration bars are 1 μm. Arrowheads indicate tight contacts between the neuron’s plasma membrane and the gold surface, *- gMμP, N- nucleus, C- cytoplasm, #- chromatin. The scale bar in **b** applies also to **c**.

**Figure 4:**
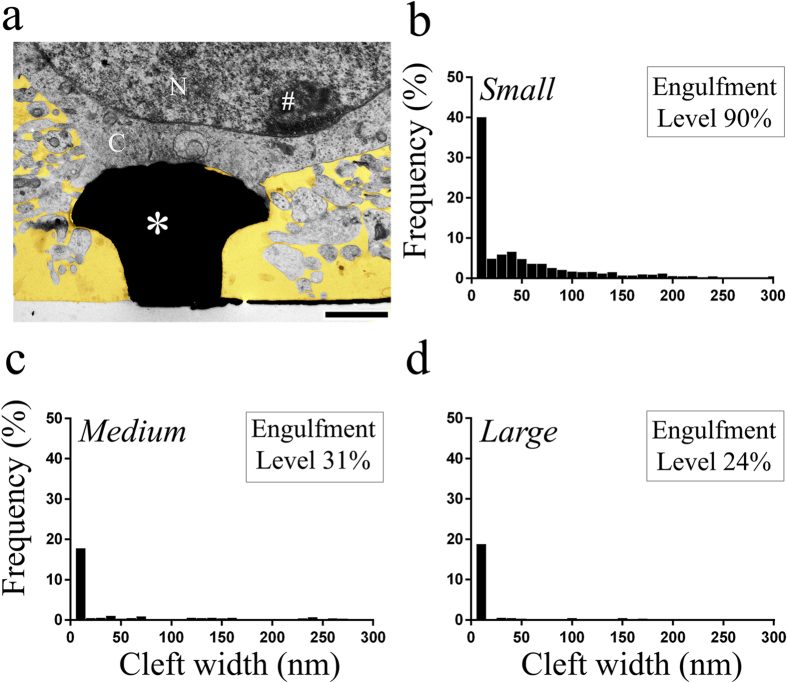
The average frequency (%) of the cleft width (nm) between the surface of gMμPs and the plasma membrane of cultured neurons. The values of the engulfment levels are indicated in the graphs. (**a**) An example of a TEM image from which the cleft width measurements were made. (**b**) Small gMμPs. (**c**) Medium size gMμPs and (**d**) Large gMμPs. (**a**). *- gMμPs, N- nucleus, C- cytoplasm, #- chromatin. For additional details see Supplementary Figs. 2–4.

To study the effects of gMμP size on the extent of their engulfment by the neurons, we characterized the neuron-gMμP interfaces by measuring the thickness (width) of the cleft formed between the neuronal plasma membrane and the gMμP surfaces (Figs. 3 and 4 and Supplementary Figs. 2–4). Since we were interested in examining how the size of the gMμP caps affects its active engulfment by the neurons, only cell bodies and large neurites that formed at least a single discernible physical contact (0–10 nm cleft) with the protruding structure were included in the quantitative analysis (Figs. 3 and 4). Measurements were made at intervals of 50 nm, perpendicular to the surface of the electrodes. The data were clustered to represent different areas of the gMμP: (a) the upper part of the mushroom cap that faces the junctional membrane of the neurons, (b) the mushroom stalk, (c) the substrate surface that corresponds to the diameter of the mushroom cap, and (d) the lower part of the mushroom cap that faces the substrate (Supplementary Figs. 2–4). When the cleft thickness between the gold surface and the cells exceeded 300 nm it was not included in the calculations of the average cleft size. The fraction (in percent) of gMμP surface with a cleft thickness smaller than 300 nm served as the “engulfment-level” parameter (Fig. 4).

The engulfment levels and average cleft thickness were 90% and 44 ± 61 nm respectively for small gMμPs (13 mushrooms, total cleft measurements 1,293), 31% and 57 ± 87 nm respectively for medium size gMμPs (10 mushrooms, total cleft measurements 1,287) and for large gMμPs the engulfment level was 24% and the cleft thickness was 22 ± 54 nm (7 mushrooms, total cleft measurements 1,161).

Examination of the tight contact formed between the neuron membrane and the upper surface of the gMμP cap revealed an identical ultrastructure independent of the cap diameter (Figs. 3 and 4 and Supplementary Figs. 2–4). The tight contacts appearing along stretches of 0.2–1 μm were interposed by short clefts of 5–10 μm. The fact that independent of the cap diameter, a 0–10 nm narrow cleft is formed between the cell’s plasma membrane and the upper surface of the gMμP suggests that the rough surface of the cap (Fig. 1) facilitates membrane adhesion to it and that this characteristic adhesion pattern is independent of mechanical forces associated with the engulfment of the gMμP stalk or the flat substrate around it (but see discussion in Santoro *et al*., 2014^33^). It is important to note that quantitative assessment of the extracellular cleft formed between the plasma membrane of living cells and an artificial substrate such as the gMμPs may reflect unknown levels of geometric artifacts (shrinking or expansion) induced by the chemical fixative used, and/or the dehydration and embedding processes^42^,^43^. A number of studies have attempted to quantify the extent of such artifacts, which has been estimated in 3D brain tissues and long-term cultures of hippocampal slices^43^,^44^. These studies indicated that the processing of tissues for TEM imaging induces tissue “shrinking “ in the range of 5–17% which in absolute terms in this study was equivalent to ± 1–4 nm^44^. It is conceivable to assume that the extent of shrinkage artifacts induced by fixative perfusion, dehydration and/or embedding of a ~10 μm thick monolayer of neurons is less than that of *in vivo* brain tissues and cultured brain slices. Although the TEM images prepared in our study did not show any signs of expansion or shrinking we estimated the possible quantitative consequences of such artifacts on the simulated neurons-gMμE electrical coupling coefficients. For instance, for measurements representing 10% shrinkage of the cleft width, the calculated coupling coefficients should be corrected by reducing the estimated coupling coefficient by 5.5–9% for a neuron-gMμE (with a cap diameter of 1.5–2.5 μm) with homogeneous cleft widths in the range of 10–100 nm (as detailed later).

We propose that the different engulfment levels of small, medium and large gMμPs reflect the limited surface area and volume of the neuronal cell bodies and neurites. Unlike many cell types, including various cell lines, primary cardiomyocytes, 2–3 day old cultured mammalian primary neurons and the *Aplysia* neurons that have been used to study the structural and functional interfacing of cells with nano and micro-protruding structures^21^,^22^,^23^,^25^,^26^,^27^,^32^,^33^,^45^, the genetic blueprint of many CNS neurons allocate an order of magnitude larger fraction of the plasma membrane to the dendrites and axonal compartments and the rest to the relatively small cell body^46^. As a result, neuron cell bodies with limited cytoskeletal machinery, very limited cytoplasmic volume, a relative large nucleus and a small membrane surface area can only follow and engulf small gMμPs, but are unable to adapt their shape and dimensions to enwrap medium and large gMμPs.

In summary, the above observations demonstrate that gMμPs with cap diameters larger than 2–2.5 μm are incompatible with the innate cell biological mechanisms that underlie the processes of gMμP engulfment by hippocampal neurons. This innate neuronal property limits the maximal gMμE cap dimensions that can be applied and thus define the physical limits of the maximal electrical coupling coefficient that can be expected from the gMμE-neurons hybrid configurations. Based on the above experimental observations and using computer simulation we next analyzed the electrical coupling coefficients that can be expected to form between cultured hippocampal neurons and engulfed gMμEs.

### Estimate of the electrical coupling coefficient between gold mushroom-shaped microelectrodes and cultured neurons

The electrical coupling coefficient (CC) is defined as the ratio between the maximal voltage amplitude of a signal recorded by the device (electrode-amplifier system) and the voltage amplitude of the signal generated across the plasma membrane of a neuron. In the simulations presented below we took the shape, dimensions and electrical properties of the gold electrodes into account as well as the level of the electrode engulfment, the frequency of the electrical signals generated by the neurons, and the passive junctional membrane properties of the neuron.

**Figure 5:**
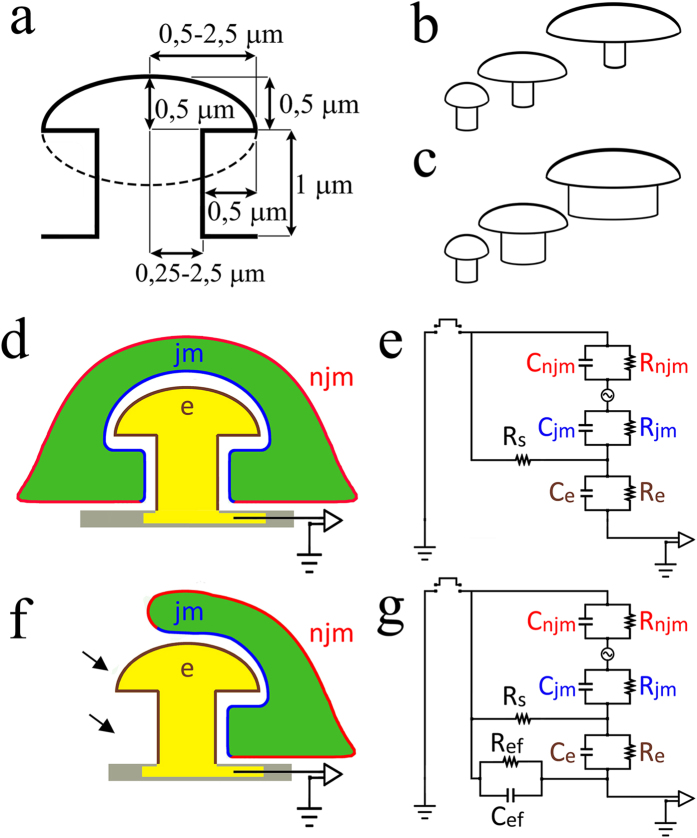
Schematic drawings of a gold mushroom-shaped microelectrode (gMμE) indicating the range of dimensions of the stalks and ellipse-shaped caps used for the simulations (a). The relationships between the dimensions of the gMμEs and the electrical coupling coefficient (CC) with neurons were simulated for two gMμE geometries: (**b**) Model A where the diameter of the stalk was maintained constant while the cap diameter was increased; and (**c**), Model B, where the diameters of the cap and stalk increased concomitantly (as shown for example in Fig. 1). (**d**–**g**) Schematic drawings and analog electrical circuits of a gMμE ((**e**), yellow) totally (**d**,**e**) and partially (**f**,**g**) engulfed by a neuron (green). Non-junctional membrane - *njm* (red), junctional membrane - *jm* (blue), electrode -*e* (yellow), arrows in (**f**) indicate the surface of gMμE exposed to the culture medium- *R*_*ef*_.

The simulations were conducted using the SPICE simulation system (Tanner EDA v.15) of analog electrical circuits. The basic configurations of the equivalent electrical circuits used are shown in Fig. 5. The circuits depict the passive membrane properties of the neuron, the electrode, the amplifier and the cleft formed between the neuron and the electrode (Fig. 5). The neurons are grown in a conducting culture medium which is grounded by an Ag/AgCl electrode. In the model the neuron’s surface area is subdivided into a non-junctional membrane (*njm*, red) that faces the grounded culture medium, and a junctional membrane (*jm*, blue) that faces the electrode. Each of these membrane compartments is represented by a resistor and capacitor in parallel *R*_*njm*_, *C*_*njm*_, *R*_*jm*_ and *C*_*jm*_ respectively. The cleft formed between the neuron and the electrode is represented by a resistor (the seal resistance-*R*_*s*_). The electrode is represented by a resistor and capacitor (*R*_*e*_, *C*_*e*_ respectively, see supplementary material for additional details).

In the model the electrical signals generated by the neurons were simulated by voltage pulses fed into the cytosol (green) which is located in the analog circuit between the *njm* and *jm* (Fig. 5). Three neuronal signals that correspond to action potentials, synaptic potentials and slow membrane oscillations were used. For the simulation of the electrical coupling of action potentials we used a sine wave of 1,000 Hz. Synaptic potentials were simulated by a 100 Hz sine wave and slow membrane oscillations by 10 Hz sine waves.

### The effects of shape and size of the gMμE on the coupling coefficients

We began by asking to what extent the size of the gMμE affects the neuron-electrode electrical CC. The simulated gMμE was constructed of a 1 μm high cylindrical stalk and a 0.5 μm high mushroom cap, shaped like a half ellipsoid transected in the plane of the long diameter (Figs. 1 and 5). To better evaluate the importance of the detailed geometry of the gMμE, we considered two modes of increasing the dimensions of the mushroom shaped microelectrode (Fig. 5b,c). In Model-A the diameter of the cylindrical stalk was maintained constant at 0.75 μm (this diameter was selected as it can be fabricated using conventional lithography) while the diameter of the mushrooms cap was increased from 1.5 to 5 μm. In Model-B the diameters of the cylindrical stalk and ellipse-shaped cap were increased concomitantly, but the cap’s diameter was always kept 1 μm larger than the stalk. This method of increasing the size mimicked the method used to fabricate the gMμPs in the biological experiments (Figs. 1, 2, 3). It should be noted that whereas the morphometric studies presented in the first part of the manuscript showed that hippocampal neurons cannot engulf gMμP with cap diameter >2–2.5 μm, we included gMμEs with cap diameters ranging from 1.5 to 5 μm in the simulation to illustrate the significant impact of the gMμE dimensions on the expected CC.

To simplify, we began with simulations assuming that the neurons adhere to the entire surface of the gMμE and to the flat substrate along an area defined by the projection of the mushroom cap onto the substrate, which corresponds to a 0.5 μm ring around the base of the stalk (Fig. 5d). The width of the cleft between the electrode surface and the neuronal plasma membrane (*d*_*j*_) was set to be homogeneous and equaled 10, 25 or 100 nm. The cleft resistivity, which corresponds to the cleft thickness, was calculated as 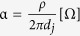 (where 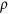 is the culture medium resistivity which equals 100 Ωcm). The simulations integrated seven parameters related to the dimensions of the gMμEs and influence the CC. These were the surface areas of the *jm* and the electrode (*e*), the corresponding values of *R*_*jm*_, *C*_*jm*_; *R*_*s*_; *R*_e_ and *C*_*e*_ (See Materials and Methods and Supplementary Material). The simulations were conducted for mushroom-shaped electrodes with cap diameters of 1.5–5 μm (Fig. 5a), and for junctional membranes with resistivities of 8 or 80 Ωcm^2^ (see Materials and Methods) and a capacitance of 1 μF/cm^2^.

**Figure 6:**
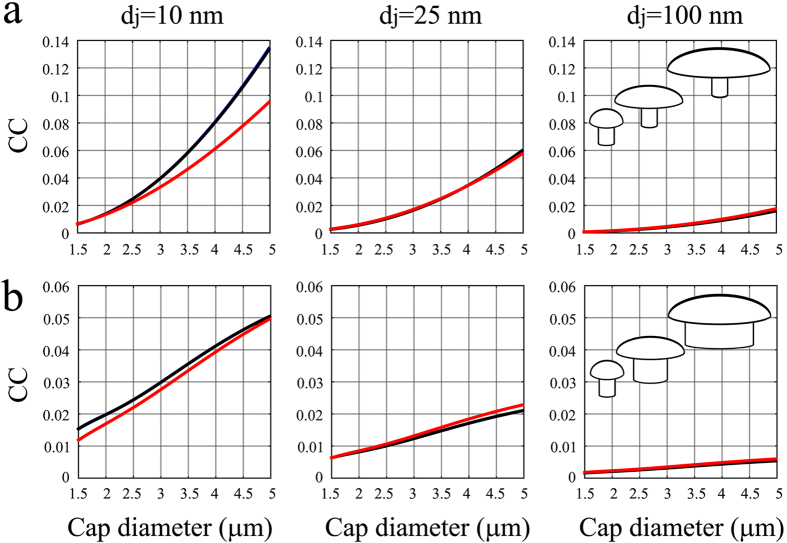
The electrical coupling coefficient between neurons and gold mushroom shaped microelectrodes (gMμEs) as a function of the mushroom cap diameters. Two modes of size change were considered (Fig. 5b,c): in (**a**) Model A, the stalk diameter was kept constant while the cap diameter was increased; in (**b**) Model B, the diameter of the mushrooms cap and stalk increased, keeping the gMμE cap diameter 1 μm larger than the stalk. The simulations were conducted assuming a homogeneous membrane-gMμE cleft thickness (*d*_*j*_) of 10, 25 or 100 nm, for three impulse frequencies depicting membrane oscillations (10 Hz), synaptic potentials (100 Hz, both oscillations and synaptic potentials are depicted by a black curve) and action potentials (1 kHz, red) and for junctional membrane resistivity (*R*_*jm*_) of 80 Ωcm^2^. All parameters related to the dimensional changes of the simulated gMμEs (*R*_*jm*_*, C*_*jm*_*; R*_*s*_*; R*_*e*_*, C*_*e*_) were integrated in the simulations using a specific membrane capacitance of 1 μF/cm^2^.

Figure 6a,b illustrate the CC as a function of the mushroom cap diameters for the two modes of gMμE geometric growth. The simulations tested three different cleft thicknesses (*d*_*j*_) of 10, 25 and 100 nm, using specific *R*_*jm*_ of 80 Ωcm^2^ and frequencies mimicking action potentials, synaptic potentials and endogenous membrane oscillations. In both gMμE model geometries, the CC declined as the cleft thickness increased (Fig. 6a,b from left to right). Comparison of the range of CC values obtained by the two gMμE models revealed that the detailed mushroom geometry affected the value of the expected CC (compare the CC values in Fig. 6a,b). Recall that the two models differed solely with regard to the diameters of the gMμE stalk: in gMμE Model-A the stalk diameter was maintained constant (0.75 μm), whereas in Model-B the stalk diameter increased with increasing cap diameter to maintain a constant relationship of a cap diameter exceeding the stalk diameter by 1 μm (see Supplementary Material). In general the comparison of the CC levels in models A and B (Fig. 6) revealed that: (a) in both models the larger the diameter of the gMμE cap, the larger the CC values. (b) In contrast, increasing the gMμE-stalk diameter decreased the effect of increased gMμE -cap diameter. (c) The larger the gMμE-neuron cleft width the smaller the CC.

Since the first part of this study established that cell biological mechanisms limit the engulfment of gMμPs to a maximal cap diameter of 2–2.5 μm, we focused the next paragraph on a description of the estimated CC values for gMμE with the maximal cap diameters that can be effectively engulfed.

Given the *R*_*jm*_ used in the simulation of Fig. 6 (80 Ωcm^2^), the expected CC levels for a totally engulfed gMμE with a cap diameter of 1.5–2.5 μm and clefts of 10, 25 or 100 nm were as follows. For Model-A (with a constant stalk diameter of 0.75 μm) 0.67–2.2%, 0.29–1.08% and 0.07–0.29% for action potentials and 0.65–2.5%, 0.29–1% and 0.065–0.26% for the synaptic potentials and membrane oscillations, respectively (Fig. 6a). For Model-B (with increasing stalk diameter from 0.5 to 1.5 μm), 1.18–2.2%, 0.63–1% and 0.45–0.5% for action potentials and 1.55–2.44%, 0.63–1% and 0.16–0.26% for the synaptic potentials and membrane oscillations, respectively (Fig. 6b).

These simulations imply that the maximal CC that can be expected using favorable theoretical conditions of a totally engulfed gMμE configuration, a cap diameter of 1.5–2.5 μm, an *R*_*s*_ produced by a homogeneous, very narrow 10 nm cleft and a *R*_*jm*_ of 80 Ωcm^2^ is at best 2.47% for the subthreshold potentials (mimicked by low frequencies of 1 and 100 Hz) and close to 2.2% for action potentials (mimicked by the 1 kHz frequency) (Fig. 6). These CC levels are sufficient for recordings of attenuated action potentials with amplitudes in the range of those reported by intracellular passive nanostructures^16^,^19^,^22^,^23^. Nevertheless, the CC levels of the slow frequencies are insufficient for recordings of synaptic potentials and membrane oscillations with source amplitudes in the range of 1–5 mV. With CC values of 2.47%, synaptic potentials of 1–5 mV will be attenuated to the noise levels of the system and below it.

As it is reasonable to assume that the innate cell biological mechanisms that limit hippocampal neurons from engulfing larger gMμE then 2–2.5 μm cannot be altered, and that a better seal resistance than that formed by a cleft of 10 nm cannot be achieved, we next examined the prospects of improving the CC by reducing the junctional membrane resistance.

### The expected impact of reduced junctional membrane resistance on the electrical coupling coefficient

It should be noted that in the proof-of-concept experimental studies using the *Aplysia* neurons-gMμE hybrid configuration our laboratory reported on the recording of action potentials with amplitudes reaching approximately 20 mV and synaptic potentials of ~2 mV. That is in these experiments the recorded signals were attenuated to approximately 25% of the input potentials^28^,^29^. To account for these high CC levels it was necessary to assume that *R*_*jm*_ should have a lower value than that directly derived from the input resistance of a neuron and the fractional area that serves as the junctional membrane^28^,^29^. To account for the high CC levels obtained in the Hai *et al*. experiments^28^,^29^ the value of *R*_*jm*_ had to be reduced by a factor of 1,000 from 100 GΩ to 100 MΩ. It was suggested that the lower *R*_*jm*_ may have been generated by the membrane curvature (formed around the gMμE) which in turn increased the density of the ionic channels within the curved patch of the junctional membrane^47^,^48^.

**Figure 7:**
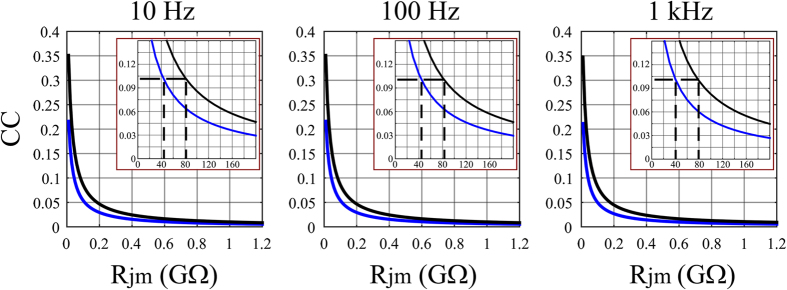
The CC as a function of the junctional membrane resistance (*R*_*jm*_) for gMμEs with cap diameters of 1.5 μm (lower curve, blue) and 2.5 μm (upper curve, black), cleft thickness of 25 nm. Inserts: enlargements of the relationships between the CC and *R*_*jm*_ in MΩ. The dashed lines in the inserts indicate that at Rjm ≤ 40–80 MΩ the CC levels reach values ≥ 10%, enabling to record subthreshold synaptic potentials.

In the simulations depicted in Fig. 6 we used an *R*_*jm*_ value of 80 Ωcm^2^ which corresponds to 1 GΩ for a gMμE with a cap diameter and stalk diameters of 1.75 and 0.75 μm respectively. To explore the range of *R*_*jm*_ values that would enable the recording of subthreshold synaptic potentials and membrane oscillations from cultured hippocampal neurons we next examined (Fig. 7) the relationships between CC and *R*_*jm*_ assuming a fully engulfed gMμE configuration, a cleft thickness of 25 nm (a more realistic cleft than 10 nm) and an electrode cap diameter of 1.5 and 2.5 μm (Model B). The simulation in Fig. 7 showed that for *R*_*jm*_ of 100 MΩ (*R*_*jm*_ resistivity of 8 Ωcm^2^) the CC of the frequencies that mimicked synaptic potentials and membrane oscillations was still below 10% and thus could hardly couple a 1 mV high synaptic potential. Nevertheless, a small additional decrease in *R*_*jm*_ from 100 MΩ to 50–80 MΩ was sufficient to increase the CC to the 10% level and thus to allow “in-cell” recordings of the electrophysiological signaling repertoire from 1 mV and above (inserts in Fig. 7). It is conceivable that such a decrease in *R*_*jm*_ may be induced by membrane curvature as discussed by Hai *et al*.^28^, by chemical functionalization of the gMμE with nano-pore forming molecules^49^,^50^, by electroporation^16^,^19^,^22^,^23^ or by molecules that lead to recruitment of ionic channels^12^,^28^.

### The expected neuron-gMμE coupling coefficients of partially engulfed gMμE

One of the expected benefits of the use of a gMμE is the improved source separation of the electrophysiological signaling with respect to classical large surface planar electrodes. Nevertheless, the transmission electron images described in the first part of this manuscript revealed that a single gMμP may be contacted or partially engulfed by a number of neuronal elements (neurites or cell bodies Figs. 2,3c and 4a). Thus, we next estimated the CC formed between neurons or neurites and gMμEs as a function of the “engulfment level” (the percentage of the electrode surface area directly in contact with the neuron) and thereby estimated the expected amplitudes that can be recorded by gMμEs that are contacted by a number of neurons.

For this simulation we used an analog electrical circuit that depicted the fraction of the surface area to which a cell membrane was adhered by two parallel RC circuits (Fig. 5f,g). One circuit represented the fraction of the gMμE in contact with the neuron’s junctional membrane and the other represented the circuit in direct contact with the grounded culture medium. For the simulation the values of *R*_*jm*_, *C*_*jm*_, and *R*_*s*_ were calculated to correspond to the fraction of the surface area that was in contact with the electrode (see Supplementary Material). The calculations of the CC as a function of the contacted surface area were made for gMμE cap diameters of 1.5 and 2.5 μm and stalk diameters of 0.5 and 1.5 μm respectively, for different *R*_*jm*_ resistivities at different frequencies. Since gold resistivity is orders of magnitude smaller than the resistances formed between the gold surface and the culture solution (~2.35 μΩcm)^51^, we simulated the occupied and free surface areas of the electrodes in an abstract manner without attempting to simulate the location of the contact between the neuron and the electrode.

**Figure 8:**
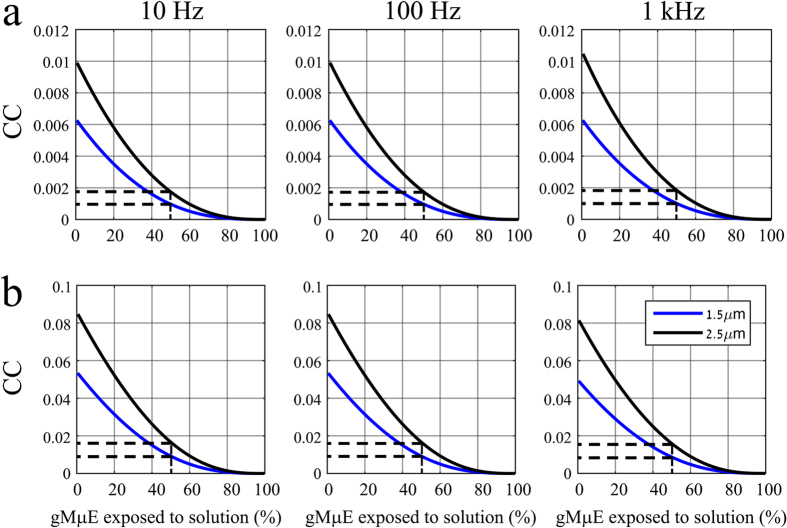
The CC as a function of the neuron-gMμE engulfment levels. The simulation was run for electrodes with cap diameters of 1.5 (lower line, blue) and 2.5 μm (upper line, black), stalk diameters of 0.75 and 1.5 μm, respectively, clefts widths of 25 nm, and *R*_*jm*_ of (**a**) 80 Ωcm^2^ and (**b**) 8 Ωcm^2^. Note that the CC of a neuron that contact 50% of a gMμE, with *R*_*jm*_ of 80 Ωcm^2^ (**a**), is too small to enable recording of any electrophysiological signals (dashed lines). On the other hand, the CC of a neuron that contacts 50% of a gMμE, with *R*_*jm*_ of 8 Ωcm^2^ (**b**), may generate small action potentials with amplitude in the range of 100–200 μV (dashed lines).

The simulation revealed that the CC declined rapidly as a function of the electrode surface exposed to the bathing solution (Fig. 8). In fact, the model predicted that a junctional membrane resistivity value of 80 Ωcm^2^ and contact area of ≤50% between a neuron and an electrode with a cleft of 25 nm does not enable recordings of any signals, since the estimated CC is as low as 0.001–0.002% (Fig. 8a). Under identical conditions, if the junctional membrane resistance was lowered to ≤8 Ωcm^2^, attenuated action potentials, but not synaptic potentials and membrane oscillation, could be detected (Fig. 8b).

This implies that in spite of its small dimension, a single gMμE can record spike activity generated by a number of neurons that form a direct contact or partially engulf a gMμE.

### The prospects of using the neuron-gMμE configuration for Recording the basic electrophysiological repertoire generated by cultured neuronal network

Given that the noise level of MEA platforms is in the range of 20–40 μV, CC values of approximately 10% for synaptic potentials and membrane oscillations (with amplitudes as small as 1 mV) and a CC of 0.5–1% for action potentials (with amplitudes in the range of 70–100 mV) may be sufficient to enable on-line acquisition of the basic electrophysiological signaling repertoire of cultured mammalian neurons.

We assume that whereas the innate cell biological limits of gMμE engulfment and gMμE-plasma membrane cleft width cannot be reduced, other parameters that influence the CC level could nevertheless be improved to reach CC levels of ≥10%. Specifically, the gMμE engulfment levels, and junctional membrane conductance could be improved by the use of: (a) Engulfment promoting peptides as discussed by Hai *et al*.^28^,^29^; (b) Pore forming molecules localized at the gMμE caps^12^,^49^,^50^; (c) Electroporation as described by number of groups^16^,^19^,^22^,^23^. Although the physical properties of the junctional membrane are dominant in defining the CC, lowering the gMμE impedance could also contribute and improve the CC. This could be achieved by applying nanometric layers of electro-active materials such as conducting polymers, carbon nanotubes, graphene and hybrid organic-inorganic nanomaterials on the electrode surface^52^. Finally, the stray capacitance of the MEA system could be improved and would also improve the CC level. In summary, it is conceivable that the use of the extracellular gMμE-neuron configuration could be used for recordings of the entire electrophysiological repertoire in the range of 1–1000 Hz with amplitudes above 1 mV.

The experimental results and simulations conducted in this study now make it possible and justifiable to proceed with the fabrication of gMμEs-MEA with maximal dimensions that can be engulfed by cultured rat hippocampal neurons, and then experimentally validate that extracellular gMμEs can record the basic electrophysiological repertoire of cultured mammalian neurons. Successful *in vitro* application of gMμEs based MEA will pave the way to applying the technology for *in vivo* use. Aside from common technological issues that affect the use of all *in vivo* MEA-platforms, specific concerns related to the *in vivo* use of gMμEs based MEA will have to be addressed. These include: (a) the mechanical stability of gMμEs to withstand sheer forces during MEA-platform insertion into the brain tissue is not known and may have to be adjusted; (b) whereas under *in vitro* conditions the neurons come into initial contact with the gMμEs through gravity, under *in vivo* conditions the initial neuron-gMμE contact will need to depend on other mechanisms. For example, by attracting the neurons towards the gMμEs by molecular signaling, or by chemical functionalization of the gMμEs surface with molecules that stabilize neuron-electrode adhesion once random contacts are formed; (c) one crucial problem in the *in vivo* use of gMμEs-MEA is the expected competition between glia and neurons for the engulfment of gMμEs. Under *in vitro* conditions the problem can be dealt with by using protocols to prepare neuronal cultures with very low glia densities. Although a solution to the issue of foreign body encapsulation by glia under *in vivo* conditions has been extensively investigated by a large number of laboratories, an effective solution is still not available.

## Materials and Methods

### Fabrication of gold mushroom shaped micro protrusion matrices

Gold mushroom-shaped micro-protrusion matrices (gMμP) were prepared on 200 μm glass wafers (AF45 Schott Glass) by means of photolithography and electroplating techniques. Briefly, the wafers were coated with an Au layer (60 nm of thermal evaporation) on top of a 10 nm Ti adhesion layer (e-gun evaporation), spin-coated with Shipley photoresist S1813 (4,000 RPM) hard baked for 10 min at 120 °C. Next, the photoresist layer was exposed to UV using a photomask with 1 μm holes with a pitch of 8 μm, 2 μm holes with a pitch of 10 μm, and 3 μm holes with a pitch of 12 μm determining the stem diameter of the gMμP. (Karl Suss MicroTec MA6 mask aligner, UV 365 nm W = 26 mW/cm^2^, exposure time: 2.7 s). Development was done using AZ726 for 35 s after which the developing agent was washed from the samples in de-ionized water. gMμPs were then electroplated using Neutronex gold plating solution at a current density of 0.2 A/cm^2^ for 45 min. The photoresist layer was then stripped using acetone and IPA. The gMμP matrices were attached to the bottom of plastic culture dishes using SylGard (Dow Corning) and dried for 48 h at 60 °C.

### Surface functionalization

Fabricated gMμP matrices were washed and sterilized by incubation in 75% ethanol for 2 h. Then, the ethanol was rinsed with double distilled water and functionalized by 0.1 mg\ml PDL (Sigma–Aldrich) and 25 μg\ml laminin (Sigma–Aldrich) in 0.1 M sodium borate, 10 mM HEPES solution (pH = 8.2) for 12 h prior to cell seeding.

### Cell culture

Rat hippocampal neurons were obtained from 17 d old embryos, as described by Kaech and Banker^37^. Briefly, a pregnant WT (Sprague Dawley) female rat was deeply anesthetized with isoflurane, the embryos removed and decapitated. The hippocampus was removed and treated with papain (Sigma–Aldrich) for 45 min, and serially triturated. Cell density at plating was 250,000–500,000 cells/ml. Cells were seeded in attachment/seeding medium [Neurobasal medium, 5% FBS, 2% B27, 1% GlutaMAX (all from Life technologies), 1% Penicillin-Streptomycin Amphotericin B Solution (Biological Industries)] on gMμP matrices (Fig. 1). 24 h after culturing, the seeding medium was replaced with serum-free maintenance/feeding medium (Neurobasal medium, 2% B27, 1% GlutaMAX, 1% Penicillin-Streptomycin Amphotericin B Solution). At 3 days *in vitro* (DIV) 2.5 μM ara-c (Sigma–Aldrich) was added to prevent glial cell proliferation. Half of the maintenance medium was replaced every 3–5 days by astroglial conditioned medium (consisting of 1/2 astroglial conditioned medium and 1/2 feeding medium). Hippocampal cultured cells were kept at 37 °C in a humidified atmosphere of 5% CO_2_. Cultures were kept up to 7–21 DIV. All procedures and experiments were approved and performed in accordance with approved guidance by the Committee for Animal Experimentation at the Institute of Life Sciences of the Hebrew University of Jerusalem.

### Immunohistochemistry and fluorescent microscopy

Cultured hippocampal cells were immunolabeled as previously described^30^ (Supplementary Fig. 1). Briefly, samples were fixed by 4% paraformaldehyde (Sigma– Aldrich) in Hank’s Balanced Salt Solution (HBSS, Biological Industries) for 30 min, washed with HBSS before membrane permeabilisation with 0.1% TritonX-100 (BDH Chemicals) in HBSS for 30 min. After washes with Tween 0.1% (J.T. Baker) in HBSS, cells were incubated for 1 h in blocking solution [BS, 2% chicken albumin (Sigma–Aldrich) in Tween 0.1%]. Then samples were incubated with primary antibodies in 1% BS overnight at 4 °C. Neurons were labeled for neuron-specific intermediate filaments with mouse anti neurofilament antibodies, and glial cells were labeled for glial fibrillary acidic protein (GFAP) with primary anti-GFAP rabbit monoclonal antibodies. The next day the samples were washed repeatedly with 0.1% Tween and incubated with secondary antibodies in 1% BS for 1 h: goat anti-mouse secondary antibodies conjugated to Cy2 (Jackson ImmunoResearch Laboratories, Inc), and goat anti-rabbit secondary antibodies conjugated to Cy3 (Life technology). Cells were counterstained with the nuclear marker DAPI (Sigma–Aldrich) for 1 h, at room temperature. Samples were washed with HBSS, and stored at 4 °C in anti-fade n-propylgallate (Sigma–Aldrich) solution in 50% glycerol until imaging. Confocal imaging of the immunolabeled cultures was done using the D-Eclipse C1 imaging system (Nikon) mounted on an Eclipse TE-2000 microscope (Nikon). Images were collected and processed using EZ-C1 software (Nikon). Scanning was done in sequential mode: red was excited with a 543 nm He–Ne laser and collected with 605 ± 75 band pass filter, green was excited with a 488 nm Argon laser and collected with a 515 ± 30 band pass filter, blue was excited with a 405 nm diode and collected with a 450 ± 35 band pass filter. Images were prepared using the open-source image analysis program ImageJ (NIH, USA) and Photoshop CS6.

### Electron microscopy

For TEM analysis, cells cultured on gMμP matrices were fixed, dehydrated and embedded in Agar 100 within the culturing dish as previously described^53^. Briefly, the neurons were fixed by 3% glutaraldehyde in a 0.1 M cacodylate buffer with a pH 7.4 for 1 h, at RT. The cells were then washed in a 0.1 M cold cacodylate buffer (pH = 7.4) (Agar Scientific, Stansted, UK). Post fixation was done with 1% osmium tetroxide (Next Chimica, Centurion, South Africa) and 0.6% K_3_Fe(CN)_6_ for 1 h, at RT. The cells were then washed in a 0.1 M cold cacodylate buffer (pH = 7.4) (Agar Scientific, Stansted, UK). Dehydration was carried out through a series of increasing concentrations of ethanol solutions, and finally the neurons were embedded in Agar 100 (Agar Scientific). Then the glass and Ti layer were etched using 39% hydrofluoric acid (for approx. 0.5 h). The Au layer was etched by a diluted Au etcher (I_2_/KI/H_2_O), leaving the gold-spine structures intact. Thereafter, the agar block, including the cells, was re-embedded in Agar 100 in a flat mold. This doubly embedded preparation was then thin-sectioned.

Measurements of cleft width from TEM images were done digitally using the image analysis program ImageJ and Photoshop CS6 (Fig. 4, Supplementary Fig. 2–4). The cleft width was measured every 50 nm perpendicularly to the gMμP surface. The significance of the differences between average cleft width values of the different areas was analyzed using student T tests and one-way ANOVAs.

For SEM analysis, cells were cultured for 6–7 d on gMμP matrices, were fixed and dehydrated as described above. Prior to the critical dry process the cultures were dehydrated by fresh 100% EtOH for 30 min. Critical point drying was conducted in liquid CO_2_ in a SAMDRI-PVT-3D apparatus (Tousimis, USA). Once dried the samples were sputtered with gold in an SPI-Module^TM^ Sputter Coater Module (SPI Supplies, USA). Images were taken with an Extra High Resolution Scanning Electron Microscopy MagellanTM 400L using an accelerating voltage of 5 kV.

### Computer simulation

Computer simulations were done using SPICE (Tanner EDA v.15), and the passive analog electrical circuit depicting a gMμE interfaced with a neuron as shown in Fig. 6 8,28,29. Calculations and graphs presentations were made using MATLAB (20014A). The main purpose of the simulations was to quantitatively characterize the relationships between the dimensions and shape of gMμE and the CC levels between the electrodes and cultured rat hippocampal neurons. The simulated gMμEs were constructed of ellipsoid-shaped caps with a constant height of 0.5 μm, and an ellipsoid cap diameter ranging from 1.5 to 5 μm (Fig. 5a). The cylindrical stalks of the gMμE were always constructed of constant 1 μm heights. Two modes of gMμE models were considered (Fig. 5a,b): in Model A the stalk diameter was kept constant at 0.75 μm while the diameter of the cap increased (Fig. 5a). In Model-B (Fig. 5b) the stalk diameter increased from 0.5 to 4 μm while the ellipsoidal cap increased from 1.5 to 5 μm. The detailed calculations of the gMμE surface are given in the Supplementary Material (Supplementary paragraph 2.1 on gMμE surface area calculations).

The resistance and capacitance of the gMμE (*R*_*e*_*, C*_*e*_), the value of the seal resistance between the electrode and the neurons (*R*_*s*_) and the dimensions of the junctional membrane surface area, its resistance and capacitance were dependent on the surface area of the gMμE (cap, stalk, and flat ring shaped area underneath the gMμE cap to which the membrane adhered) and the engulfment level of the electrode by a neuron. These parameters were calculated in the following manner:

### gMμE resistance and capacitance

The impedance of the gMμE itself is negligible with respect to the impedance created at the interface between the ionic solution and the gMμE. For this reason the values of *R*_*e*_ and *C*_*e*_ used here refer to the resistance and capacity of the electrode/ionic solution interface. To define *R*_*e*_ and *C*_*e*_ for gMμE of different sizes and shapes we calculated the values of the electrode resistivity (Ωcm^2^) and the specific electrode capacitance (F/cm^2^). This was done by first measuring the impedance of freshly fabricated gMμEs with a cap diameter of 1.75 μm, a height of 0.5 μm and a stalk diameter of 0.75 μm using an HP 4284A Precision RLC meter, at 1 KHz, at room temperature, in 0.9% NaCl solution, between individual gMμEs and a counter Ag/AgCl electrode. The average electrode resistance was 3.5 MΩ and the capacitance was 5.1 PF (Supplementary material paragraph 2.2). These measurements were used to calculate an average electrode surface resistivity value of 0.28 Ωcm^2^ and an average specific capacitance of 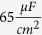. For each size and shape of the gMμE, the electrode resistance and capacitance was calculated relative to the surface area of a gMμE with a measured resistance value as described above. Formally,


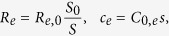


where *R*_*e,0*_ – electrode resistance for a gMμE with the above mentioned dimensions, 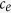
– electrode capacitance, 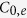
- electrode specific capacitance, *S*_*0*_– Surface area for this gMμE, *S* – Surface area as a function of the dimensions. The electrode capacitance was calculated in a similar manner.

### Calculation of the seal resistance (*R*_*s*_)

The seal resistance between the neuronal membrane and the gMμE was calculated by integration of infinitesimal resistors connected in series along the cleft path (between the electrode and the cell membrane) from the center top of the gMμE cap to its stalk-base and along a 0.5 μm (ring shape) flat substrate that surrounds the stalk base (see Supplementary material paragraph 2.3 on the calculation of the seal resistance). For the calculations we assumed that the current generated by the neurons flowed through homogeneously distributed ion channels along the entire junctional plasma membrane (see Supplementary Fig. 5). Note that in earlier studies we assumed for the calculation of *R*_*s*_ that the current flowed from a single point source at the center top of the gMμE cap. Since the direction of the current flow in the present model was from the mushroom cap along the stalk, through the small flat ring-shaped region around the stalk, each infinitesimal resistor only affects the fraction of current generated “above” it. The effective contribution of the “infinitesimal resistors” to the seal resistance is therefore given by the naive calculation of resistance, multiplied by a weight function defined as the ratio between the electrode area above it and the total area of the electrode.

Using the calculated surface area of the gMμE, we next calculated the corresponding values of: (a) the junctional membrane resistance and capacitance, (b) the seal resistance assuming different cleft thickness and the specific resistivity of the culture medium (100 Ωcm). For the simulations we used the following parameters: (1) the non-junctional membrane resistance of 100–250 MΩ (*R*_*njm*_) has been measured in a large number of publications^54^,^55^. The values of the junctional membrane resistance (*R*_*jm*_) were derived by dividing the resistivity of the non-junctional membrane by the junctional membrane surface area. These values correspond to plasma membrane resistances of 100 MΩ, 1 GΩ, and 100 GΩ for a gMμE with a cap diameter of 1.75 μm and a stalk diameter of 0.75 μm. Based on the earlier results in Hai *et al*.^28^,^29^ *R*_*njm*_ was estimated two different values of membrane resistivity that represent three estimated values of channel densities in the plasma membrane which curves around the electrodes. These were defined as 8 Ωcm^2^ (corresponding to 100 MΩ for *R*_*jm*_ that faces a gMμE with a cap diameter of 1.75 μm and a stalk diameter of 0.75 μm) and 80 Ωcm^2^ (corresponding to a 1 GΩ *R*_*jm*_ that faces a gMμE with a cap diameter of 1.75 μm. (3) The estimated junctional membrane capacitance (*C*_*jm*_) was calculated for any given contact surface area between the simulated cells and the simulated gMμE and the universal value of the specific membrane capacitance (1 μF/cm^2^). (4) The voltage pulses that simulated action potentials, synaptic potentials and membrane oscillations were delivered to the simulated neurons between the junctional (*jm*) and non-junctional membranes (*njm*). (5) An amplifier input capacitance of 8 pF and a resistance of 100 GΩ were used in all simulations.

### The coupling coefficients

(CC) were calculated as the voltage ratios between the amplitudes of the simulated gMμE and the potential generated within the simulated cell.

## Additional Information

**How to cite this article**: Ojovan, S. M. *et al*. A feasibility study of multi-site, intracellular recordings from mammalian neurons by extracellular gold mushroom-shaped microelectrodes. *Sci. Rep*. **5**, 14100; doi: 10.1038/srep14100 (2015).

## Supplementary Material

Supplementary Information
