# Supplementary material for: A feasibility study of multi-site,intracellular recordings from mammalian neurons by extracellular gold mushroom-shaped microelectrodes
Source: Sci Rep. 2015 Sep 14;5:14100. doi: 10.1038/srep14100 (PMC4568476; doi:10.1038/srep14100)
Supplement: Supplementary Information [file srep14100-s1.pdf]

A feasibility study of multi-site, intracellular recordings from mammalian neurons by extracellular gold mushroom-shaped microelectrodes

Silviya M. Ojovan[1], Noha Rabieh[1], Nava Shmoel[1], Hadas Erez[1], Eilon Maydan[1], Ariel Cohen[2] and Micha E. Spira[1*]

1 Supplementary Figures 1-4

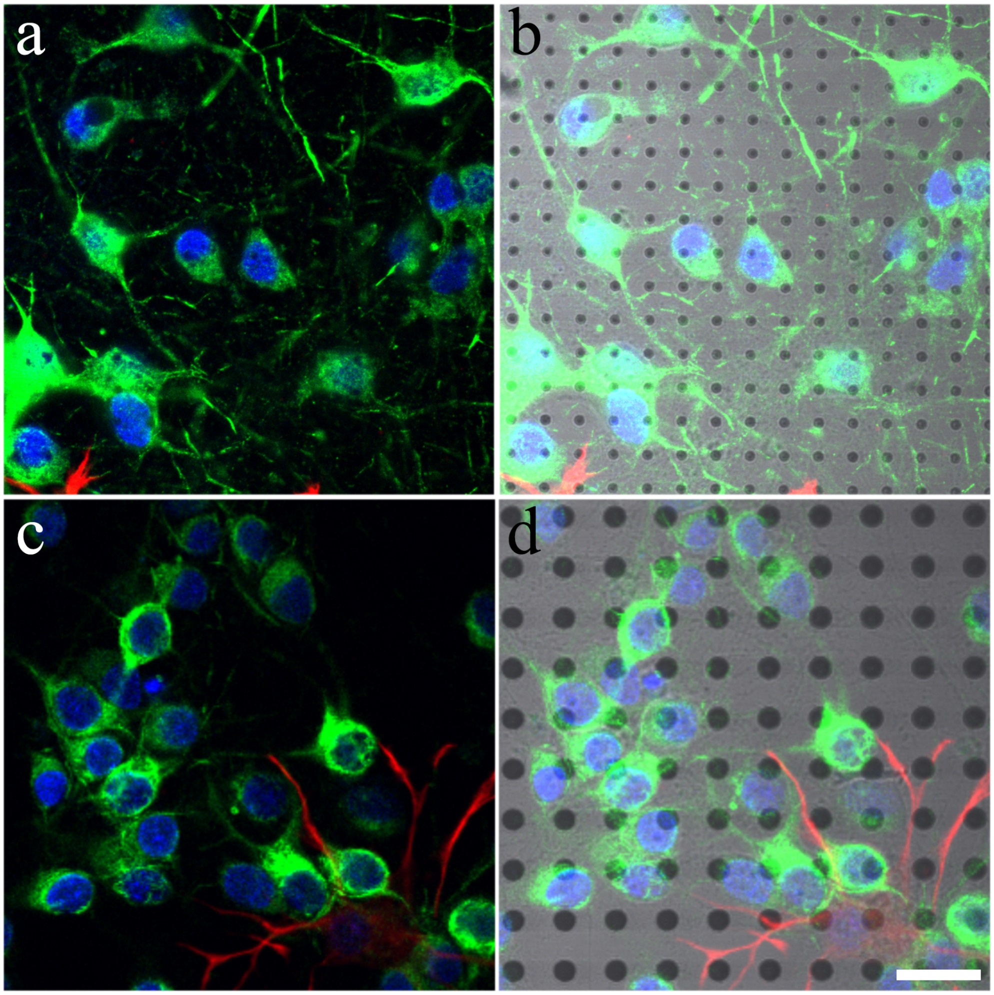

Supplementary Figure 1. . Confocal images of primary hippocampal neurons grown on matrices of gold mushroom-shaped protruding microstructures (gMµPs) for 7 days. Note that the density of glial cells (red) is low (3%) increasing the probability of the neurons (green) to form direct contacts with the gMµPs. (a) and (b) neurons grown on matrices of small gMµPs, (c) and (d) neurons grown on large gMµPs. (a) and (c) images of immunolabeled cells only, (b) and (d) superimposed light and fluorescent microscope images of the gMµPs matrices and the fluorescently labeled cells. Calibration bar - 20µm.

A feasibility study of multi-site, intracellular recordings from mammalian neurons by extracellular gold mushroom-shaped microelectrodes

Silviya M. Ojovan[1], Noha Rabieh[1], Nava Shmoel[1], Hadas Erez[1], Eilon Maydan[1], Ariel Cohen[2] and Micha E. Spira[1*]

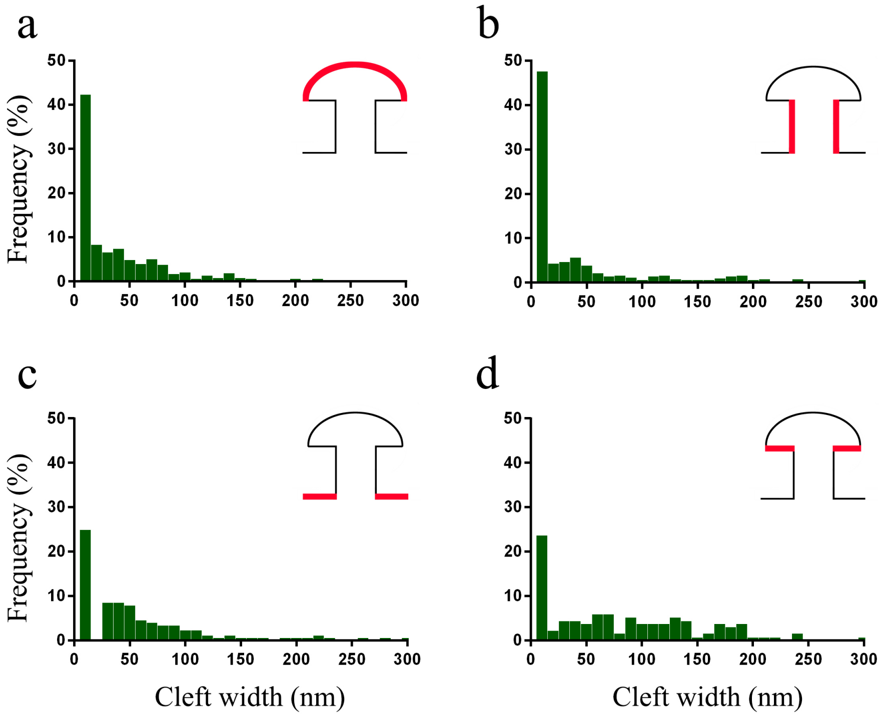

Supplementary Figure 2. The frequency (%) of different cleft widths (nm) formed between the surface of small gMµPs and the neuron's plasma membrane. The cleft width was measured in 4 different compartments (marked in red) of the gMµPs: (a) the cap surface that faces the junctional membrane; (b) the surface ot the stalk; (c) the substrate; and (d) the surface under mushroom cap.

A feasibility study of multi-site, intracellular recordings from mammalian neurons by extracellular gold mushroom-shaped microelectrodes

Silviya M. Ojovan[1], Noha Rabieh[1], Nava Shmoel[1], Hadas Erez[1], Eilon Maydan[1], Ariel Cohen[2] and Micha E. Spira[1*]

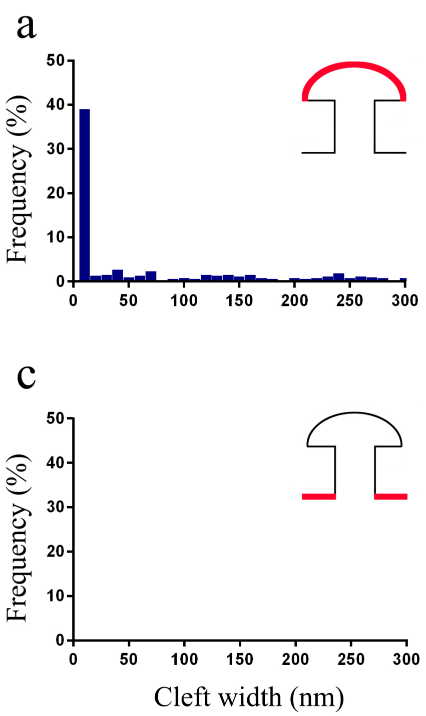

Supplementary Figure 3. The same as Supplementary Figure 2 but for a medium size gMμPs.

A feasibility study of multi-site, intracellular recordings from mammalian neurons by extracellular gold mushroom-shaped microelectrodes

Silviya M. Ojovan[1], Noha Rabieh[1], Nava Shmoel[1], Hadas Erez[1], Eilon Maydan[1], Ariel Cohen[2] and Micha E. Spira[1*]

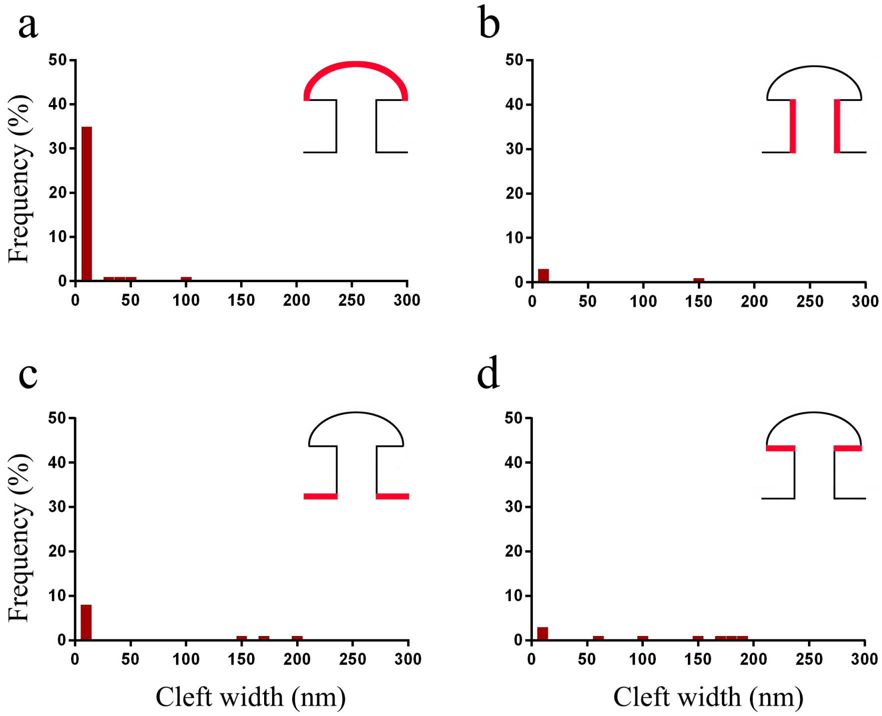

Supplementary Figure 4. The same as Supplementary Figure 2 but for a large size gMµPs

2 Calculation of parameters used in the simulations

To define the electrical properties of the gMμE and the junctional membrane, we first calculated the gMμE surface area (2.1) and then derived their electrical characteristics (2.2).

2.1 gMμE surface area

The surface area of a gMμE is the sum of the hemi-ellipsoid cap (the upper surface of the cap), the flat part of the gMμE under the mushroom cap and the stalk (Supplementary Figure 2.). The surface area of an ellipsoid is given approximately by-

$$S \approx 4\pi \left(\frac{(ab)^{1.6} + (ac)^{1.6} + (bc)^{1.6}}{3} \right)^{\frac{1}{1.6}} \tag{1}$$

where a,b,c are the ellipsoid semi-axis lengths. Therefore the surface area of half of an ellipsoid with two identical semi-axis lengths r_{cap} (the gMμE cap radius),

and with the third being h_{cap}(the height of the cap) is-

$$S \approx \frac{1}{2} \cdot 4\pi \left(\frac{(r_{cap} \cdot r_{cap})^{1.6} + (r_{cap} h_{cap})^{1.6} + (h_{cap} r_{cap})^{1.6}}{3} \right)^{\frac{1}{1.6}} \qquad (2)$$

The simulations assumed constant h_{cap} of $0.5\mu m$.
The flat area at the bottom of the gMμE cap is given by subtraction of the area occupied by the stalk with a radius of r_{stalk} from the the flat area at the botom of the gMμE cap with radius r_{cap}.

$$S_{bottom} = \pi r_{cap}^2 - \pi r_{stalk}^2 = \pi \left(r_{cap}^2 - r_{stalk}^2 \right) \qquad (3)$$

The surface area of the stalk is given by-

$$S_{stalk} = 2\pi r_{stalk} h_{stalk} \qquad (4)$$

The sum of these is-

$$\begin{aligned} S_{\text{gM}\mu\text{E}} &= S_{cap} + S_{bottom} + S_{stalk} \qquad (5) \\ &= 2\pi \left(\frac{r_{cap}^{3.2} + 1.65 \times 10^{-10} \cdot r_{cap}^{1.6}}{3} \right)^{\frac{1}{1.6}} \\ &+ \pi \left(r_{cap}^2 - r_{stalk}^2 \right) + 2\pi r_{leg} h_{leg} \end{aligned}$$

2.2 Capacitance and conductance of the gMμE and the junctional membrane

Using impedance measurement of individual gMμEs ($r_{cap} \approx 1.75\mu m$, $h_{cap} \approx 0.5\mu m$, $r_{stalk} \approx 0.75\mu m$, $h_{stalk} \approx 1\mu m$) at $1KHz$ in an ionic solution of 0.9% NaCl, average values of a constant phase element with a resistance of $3.5M\Omega$ and a capacitance of $5.1pF$ in series were extracted. Impedance spectrum measurments indicated the pure capacitive characteristics of the gMμE with an impedance that follows $Z \propto \frac{1}{f}$ (where f is the frequency in the range of 1 to 100kHz). Hence the complex nature of the CPE could be neglected for all the relevant frequencies and be presented as a simple passive element. Previous studies have shown the existence of a resistor in parallel to the CPE mentioned above with much greater impedance with respect to the CPE, which also makes it negligible. Using these values and the calculated surface area we extracted a specific capacitance value of the gMμE-ionic solution interface of $65\frac{\mu F}{cm^2}$ and specific resistivity of $0.28\Omega cm^2$. For the specific capacitance of the membrane we used a value of $1\frac{\mu F}{cm^2}$. Recall that different junctional membrane resistivities were applied for the simulations. Both the junctional membrane and the gMμE capacitances are obtained by multiplying the suitable specific capacitance by the gMμE surface area, whereas the junctional membrane and the gMμE resistance are obtained by dividing the suitable specific resistance by the gMμE surface area.

A feasibility study of multi-site, intracellular recordings from mammalian neurons by extracellular gold mushroom-shaped microelectrodes

Silviya M. Ojovan[1], Noha Rabieh[1], Nava Shmoel[1], Hadas Erez[1], Eilon Maydan[1], Ariel Cohen[2] and Micha E. Spira[1*]

2.3 Seal Resistance (R_s)

The seal resistance formed between the gMμE and the membrane of the cell engulfing it (denoted R_s) is calculated by adjusting the basic formula of resistance of a cuboid given by-

$$R = \rho \frac{L}{A} \tag{6}$$

where: ρ - Specific resistance, L - Length of the resistor, A - cross-sectional area through which the electrical current flows. R_s represents the sum of resistances generated along the surface of a gMμE. The following sub-paragraphs deal with the seal resistance generated along the different parts of the gMμE.

2.3.1 gMμE cap (upper part)

The simulations were conducted for gMμE with an ellipsoid shaped cap (**Supplementary Figure 5**). To calculate the electrical resistance formed by the cleft along the ellipse shaped cap, the path from the top of the cap towards its flat part was divided into infinitesimal summed resistors. To that end, we calculated the value of each infinitesimal resistor surface area through which the current flows and its length. The length element is given by-

$$dl = \sqrt{a^2 \cos^2(t) + b^2 \sin^2(t)}dt \tag{7}$$

For ellipsoid with major semi axis a and minor semi axis b. The resistor cross sectional area is given by-

$$A(t) \approx 2\pi d_j a \sin(t) \tag{8}$$

Therefore,

$$R_s = \int_{\theta_0}^{\frac{\pi}{2}} \frac{\sqrt{a^2 \cos^2(t) + b^2 \sin^2(t)}}{2\pi d_j a \sin(t)} dt = \alpha \int_{\theta_0}^{\frac{\pi}{2}} \frac{\sqrt{a^2 \cos^2(t) + b^2 \sin^2(t)}}{a \sin(t)} dt \tag{9}$$

Where: $\alpha = \frac{1}{2\pi d_j}$. The value of the integral above (**Supplementary equation.** (9)) grows rapidly as $\theta_0 \to 0$, implying that the seal resistance generated at the top of the cap tends toward infinity. However, assuming a homogoneous distribution of ionic channels across the membrance, the effect of the large resistance at the top of the cap is considerably reduced. In order to normalize this effect, we used a weight function, $W(t)$, which represents the ratio between the surface area of the gMμE(between the top of the gMμE and the differential resistance element) affected by the infinitesimal resistor that is currently summed in the integral, and the surface area of the whole gMμE, thus giving a normalized value to the infinitesimal resistor. Supplementary equation (9) then becomes-

$$dR_s = \frac{\sqrt{a^2 \cos^2(t) + b^2 \sin^2(t)}}{2\pi d_j a \sin(t)} W(t) \tag{10}$$

A feasibility study of multi-site, intracellular recordings from mammalian neurons by extracellular gold mushroom-shaped microelectrodes

Silviya M. Ojovan[1], Noha Rabieh[1], Nava Shmoel[1], Hadas Erez[1], Eilon Maydan[1], Ariel Cohen[2] and Micha E. Spira[1*]

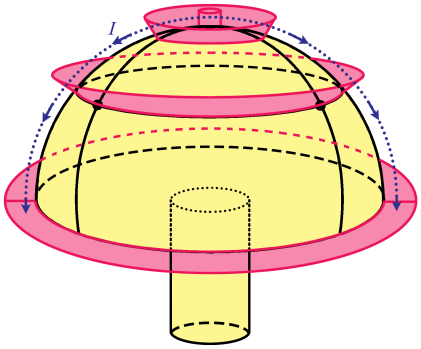

Supplementary Figure 5. schematically represents the current flow over thegMμE cap (presented in yellow), and the infinitesimal resistive elements (depicted in pink) along the current path towards the cap bottom. I - The current along the gMμE cap

2.3.2 Disc

Even though the current flows radially both on the bottom of the cap and around the base of the stalk, These are two different cases and therefore the weight function to be used is different.

The flat part of the cap

$$dR_s = \rho\frac{dr}{2\pi r d_j}\cdot\frac{\pi\left(r_{cap}^2-r^2\right)+S_{cap}}{S_{jm}} \tag{11}$$

$$\begin{aligned} R_s &= \frac{\alpha}{S_{jm}}\int_{r_{stalk}}^{r_{cap}}\left(\frac{\pi r_{cap}^2+S_{cap}}{r}-\pi r\right)dr \\ &= \frac{\alpha}{S_{jm}}\left[\frac{1}{2}\pi\left(r_{stalk}^2-r_{cap}^2\right)+\left(\pi r_{cap}^2+S_{cap}\right)\ln\left(\frac{r_{cap}}{r_{stalk}}\right)\right] \end{aligned} \tag{12}$$

Substrate ring around the stalk to which the membrane adhere

$$dR_s = \rho\frac{dr}{2\pi r d_j}\cdot\frac{\pi\left(r^2-r_{stalk}^2\right)+S_{cap}+S_{stalk}+S_{bottom}}{S_{jm}} \tag{13}$$

$$\begin{aligned} R_s &= \frac{\alpha}{S_{jm}}\int_{r_{stalk}}^{r_{cap}}\left(\pi r+\left(S_{cap}+S_{stalk}+S_{bottom}-\pi r_{stalk}^2\right)\frac{1}{r}\right)dr \\ &= \frac{\alpha}{S_{jm}}\left[\left(S_{cap}+S_{stalk}+S_{bottom}-\pi r_{stalk}^2\right)\ln\left(\frac{r_{cap}}{r_{stalk}}\right)+\frac{1}{2}\pi\left(r_{cap}^2-r_{stalk}^2\right)\right] \end{aligned} \tag{14}$$

2.3.3 Cylinder

Using the same logic above for the stalk which is cylinder shaped,

$$dR_s = \rho \frac{dh}{2\pi r_{stalk} d_j} \cdot \frac{2\pi r_{stalk}\left(h_{stalk} - h\right) + S_{cap} + S_{bottom}}{S_{gM\mu E}} \tag{15}$$

Integration gives-

$$\begin{aligned} R_s &= \frac{\alpha}{S_{jm} r_{stalk}} \int\limits_0^{h_{stalk}} \left(2\pi h_{stalk} r_{stalk} + S_{cap} + S_{bottom} - 2\pi r_{stalk} h\right) dh \qquad (16) \\ &= \frac{\alpha}{S_{jm} r_{stalk}} \left(\left(2\pi h_{stalk} r_{stalk} + S_{cap} + S_{bottom}\right) h - \pi r_{stalk} h^2\right)\Big|_0^{h_{stalk}} \\ &= \frac{\alpha}{S_{jm} r_{stalk}} \left[\left(2\pi r_{stalk} h_{stalk}^2 + S_{cap} h_{stalk} + S_{bottom} h_{stalk}\right) - \pi r_{stalk} h_{stalk}^2\right] \\ &= \frac{\alpha}{S_{jm} r_{stalk}} \left[\pi r_{stalk} h_{stalk}^2 + S_{cap} h_{stalk} + S_{bottom} h_{stalk}\right] \end{aligned}$$

3 Partial Engulfment

For the case of partial engulfment of the gMμE, values of R_{jm}, C_{jm}, R_e, C_e and R_S were calculated as follows: If we denote the values of a fully engulfed gMμE by $R_{jm,0}, C_{jm,0}, R_{e,0}, C_{e,0}, R_{s,0}$, and the fraction of it directly exposed to the bathing solution by f, than these effective values as function of f are given by:

$$\begin{aligned} R_{jm} &= \frac{R_{jm,0}}{1-f} \qquad (17) \\ R_e &= \frac{R_{e,0}}{1-f} \\ C_{jm} &= C_{jm,0}\left(1-f\right) \\ C_e &= C_{e,0}\left(1-f\right) \\ R_s &= R_{s,0}\left(1-f\right) \end{aligned}$$

and the values of $R_e^*, C_{e,0}^*$, representing the fraction of the electrode dire and the values of $R_e^*, C_{e,0}^*$, representing the fraction of the electrode directly facing the bathing solution are given by:

$$\begin{aligned} R_{ef} &= \frac{R_{e,0}}{f} \qquad (18) \\ C_{ef} &= R_{e,0} \cdot f \end{aligned}$$

It is important to note that R_s is considered to be directly proportional to the gMμE surface area; i.e.,the shape of the gMμE is not exactly taken into account, only the percentage of exposure to the solution.

A feasibility study of multi-site, intracellular recordings from mammalian neurons by extracellular gold mushroom-shaped microelectrodes

Silviya M. Ojovan[1], Noha Rabieh[1], Nava Shmoel[1], Hadas Erez[1], Eilon Maydan[1], Ariel Cohen[2] and Micha E. Spira[1*]

4 References

William D. Callister, David G. Rethwisch. in *Materials Science and Engineering: An Introduction, 8th edn,* Ch. 18.8, 733 (John Wiley, 2010).
